# Supplementary material for: Diabetes service decentralization to primary healthcare unit in Tigray, Ethiopia: A pilot study
Source: PLoS One. 2025 May 7;20(5):e0320296. doi: 10.1371/journal.pone.0320296 (PMC12058178; doi:10.1371/journal.pone.0320296)
Supplement: Attachment — Submitted filename: Review for PLoS One.docx [file pone.0320296.s002.docx]

Review for PLoS One

Sun 1 Sept 2024

Title: Diabetes service decentralize to Primary health care unit; A pilot study

Short title

Healthcare unit, not health case unit.

Abstract

Introduction

“Ethiopia has 2.57 million adults with diabetes (5.2 % of adult population) and 4.9 million adults with prediabetes. This is estimated to be the largest diabetes population in Sub-Saharan Africa. 76% do not even know that they have diabetes.” Please put references for both statements.

“Studies have shown that decentralized diabetes care has effect of diabetes morbidity and mortality and has good long term and short-term outcome. In addition, there was no significant difference in conducting necessary diabetes test, risk factors assessments and service delivery cares among referral and primary care health facilities (4-6).”

This is not clear. Which studies? Which countries or settings? Please rewrite this section.

Study setting

- It is not clear about the patients the authors moved from Ayder to Hagereselam. The authors did not explain, for example, if the patients moved from Ayder are diabetic patients who are residents of Hagereselam and its vicinity.
- Please mention Hagereselam’s elevation, demographics etc.
- Please describe the steps you followed to discuss with patients to transfer them from Ayder to Hagereselam. How many patients have you contacted to get 67 willing patients? How many patients form Hagereselam’s vicinity had follow in Ayder? How many agreed to be moved to Hagereselam? How did you address their concerns? Unless you explain these things, we cannot know if the patients were moved voluntarily or coerced.

Results

- In the study setting you mentioned that newly diagnosed patients from Hagereselam and veteran patients from Ayder Hospital were the study population. You implied that for this purpose, healthcare professionals were trained to diagnose new diabetic patients. However, in the results section, you mentioned that only those transferred from Ayder were followed. Please clarify this.
- It is not clear how you involve patients from Ayder for comparison. You have not mentioned in your methods section about patients selected in Ayder for comparison. However, in your results section, you mentioned that you compared your 3 months lab results with randomly selected patients. This needs an explanation in your methods section. Who is this patient population you are comparing with your study population? Are you talking about the same patients you are using for pilot, or you used the same patients’ prior records? If the case is the later, it is not right as you are neglecting temporal effects.

Discussion

- You are implying that patients are satisfied. How did you assess their satisfaction levels? What model have you used to measure it?

Limitations

- Why did the authors decide to cut short/abort the study? It would have made sense to continue it to get a good picture even after the Cessation of Hostilities agreement. As they have clearly stated, the study does not show the whole picture. For example, though not very clearly stated, they mainly relied on veteran patients from Ayder to conclude. Based on this study’s findings, we cannot conclude that new patients are safe to be treated at the primary healthcare units.
